# Early Treatment of Acute Myocardial Infarction with Melatonin: Effects on MMP-9 and Adverse Cardiac Events

**DOI:** 10.3390/jcm11071909

**Published:** 2022-03-30

**Authors:** Alberto Domínguez-Rodríguez, Daniel Hernández-Vaquero, Pedro Abreu-González, Néstor Báez-Ferrer, Rocío Díaz, Pablo Avanzas, Fedor Simko, Virginia Domínguez-González, Ramaswamy Sharma, Russel J. Reiter

**Affiliations:** 1 Servicio de Cardiología, Hospital Universitario de Canarias, 38010 Santa Cruz de Tenerife, Spain; nestor.baez@hotmail.com; 2 Facultad de Ciencias de la Salud, Universidad Europea de Canarias, 38200 Santa Cruz de Tenerife, Spain; 3 CIBER de Enfermedades Cardiovasculares (CIBERCV), 28029 Madrid, Spain; 4 Área del Corazón, Hospital Universitario Central de Asturias, 33011 Oviedo, Spain; dhvaquero@gmail.com (D.H.-V.); diazmendezro@gmail.com (R.D.); avanzas@gmail.com (P.A.); 5 Instituto de Investigación Sanitaria del Principado de Asturias, 33011 Oviedo, Spain; 6 Departamento de Fisiología, Facultad de Medicina, Universidad de La Laguna, 38200 Santa Cruz de Tenerife, Spain; pabreugonzalez@gmail.com; 7 Departamento de Medicina, Universidad de Oviedo, 33003 Oviedo, Spain; 8 Institute of Pathophysiology, Faculty of Medicine, Comenius University, 81108 Bratislava, Slovakia; fedor.simko@fmed.uniba.sk; 9 School of Medicine, Facultad de Ciencias de la Salud, Universidad de La Laguna, 38200 Santa Cruz de Tenerife, Spain; virgi2002@hotmail.es; 10 Department of Cell Systems and Anatomy, Joe R. and Teresa Lozano Long School of Medicine, UT Health San Antonio, San Antonio, TX 78229, USA; sharmar3@uthscsa.edu

**Keywords:** melatonin, acute myocardial infarction, primary percutaneous coronary intervention, major cardiovascular events, MMP-9

## Abstract

Background: Matrix metalloproteinase-9 (MMP-9) is crucial in tissue remodeling after an adverse cardiac event. In experimental studies, melatonin has been found to attenuate MMP-9 activation. The present study assessed the effects of systemic melatonin administration on the prognosis of patients with acute myocardial infarction (AMI) successfully treated with primary percutaneous coronary intervention, and to examine the effects on MMP-9 levels. Methods: We conducted a randomized controlled trial, enrolling patients who underwent primary percutaneous coronary intervention due to AMI. They were assigned to two groups for melatonin or placebo. The primary endpoint was a combined event of mortality and heart failure readmission at 2 years. The secondary endpoint was the levels of MMP-9 after the percutaneous coronary intervention. Results: Ninety-four patients were enrolled, 45 in the melatonin group and 49 in the control group. At 2 years of follow-up, 13 (13.8%) patients suffered the primary endpoint (3 deaths and 10 readmissions due to heart failure), 3 patients in the melatonin group and 10 in the placebo group. The difference in the restricted mean survival time was 87.5 days (*p* = 0.02); HR = 0.3 (95% CI 0.08–1.08; *p* = 0.06); Log-rank test 0.04. After controlling for confounding variables, melatonin administration reduced MMP-9 levels to 90 ng/mL (95% CI 77.3–102.6). Conclusions: This pilot study demonstrated that compared to placebo, melatonin administration was associated with better outcomes in AMI patients undergoing primary percutaneous coronary intervention.

## 1. Introduction

The past 35 years have seen intense research to find drugs to reduce myocardial ischemia-reperfusion injury (IRI) [1]. Several preclinical studies have reported encouraging findings. However, with few exceptions, results have been disappointing or contradictory when candidate drugs have reached the clinical phase [1]. One of the reasons for this failure is the complexity of IRI, involving various mediators and signaling pathways simultaneously, which hinders the effectiveness of more selective targeted actions on a single component [2].

The IRI causes numerous collateral reactions in tissues which culminate in the alteration of essential molecules and organelles in a number of cells, including components of the coronary endothelium and myocardium, with the recruitment of circulating blood elements such as leukocytes and platelets. During transient ischemia and the reperfusion period, many cells produce oxygen by-products that are toxic to the heart. In fact, partially reduced oxygen metabolites and the enhanced activation of matrix metalloproteinases (MMP) cause much of the cardiac damage that is sustained during IRI [3].

Solid evidence exists that melatonin influences the cardiovascular system [4,5,6,7]. A post hoc analysis investigated whether the melatonin effect is time dependent, i.e., from symptom onset to artery opening in patients with AMI treated with primary angioplasty [8]. The hypothesis-generating analysis found a relation between treatment effect and ischemia time and the authors concluded that melatonin administration within 3 h of ischemia onset reduces myocardial infarct size by approximately 40% [8]. We hypothesize that early treatment with melatonin protects the infarcted heart by reducing extracellular MMP deposition and, therefore, could lead to a reduced rate of adverse cardiac events. The aims of this study were: (a) to assess whether administration of intravenous melatonin starting immediately before reperfusion improves clinical outcomes; (b) to investigate the effect of melatonin on MMP-9 levels in patients with AMI successfully treated with primary percutaneous coronary intervention.

## 2. Methods

### 2.1. Study Population

Prospective, randomized, open-label study conducted in patients presenting with ST-segment elevation AMI who received successful primary percutaneous coronary intervention from September 2017 until December 2018. As per protocol, melatonin had to be initiated within 3 h of symptom onset. Main exclusion criteria were: age ≤ 18 or ≥ 85 years, severe renal failure (estimated glomerular filtration rate < 30 mL/min/1.73 m^−2^), hepatic failure (Child–Pugh class B or C), known malignancy, ventricular fibrillation, cardiac arrest, cardiogenic shock as presenting symptom, previous myocardial infarction. The protocol was approved by the institutional review board and was implemented in accordance with the Declaration of Helsinki. All patients provided informed consent.

Eligible patients were equally randomized for treatment with placebo (50 mL physiological saline) or intravenous melatonin 0.24 mg/mL (12 mg in 50 mL). The treatment with placebo or melatonin was delivered as an intravenous infusion for 60 min, starting immediately before primary percutaneous coronary intervention. Of the total cohort, all the patients received solely drug-eluting stents. In addition, antiplatelet drugs, beta-blockers, anticoagulants, statin therapy, and angiotensin-converting enzyme inhibitors were prescribed for all eligible patients as recommended by recent guidelines [9].

Follow-up data were collected using hospital source data as well as all medical records. Contact with patient or family and physicians from other centers was performed when needed.

### 2.2. Blood Sample Collection

Blood samples were drawn just after completion of the primary percutaneous coronary intervention. Serum samples for MMP-9 levels assessment were collected and aliquoted into several tubes and stored at −80 °C until analysis. Sandwich enzyme immunoassay was performed for measuring concentrations of serum MMP-9 using commercially available kit with monoclonal antibody against the substance [10], according to the manufacturer’s instructions (Calbiochem, San Diego, CA, USA). Briefly, an antibody specific for the human MMP-9 protein was immobilized on the surface of microtiter wells, followed by addition of the sample to the wells. After incubation and washing, a biotin-conjugated monoclonal anti-MMP-9 antibody was added. Following incubation, unbound biotin-conjugated anti-MMP-9 was removed by washing, followed by addition of streptavidin horseradish peroxidase (HRP) and incubation. After washing, substrate solution reactive with HRP was added to the well (tetra-methylbenzidine, TMB). The reaction was terminated by the addition of 2.5 N sulfuric acid and absorbance was measured at 450 nmin on a microplate spectrophotometer (Spectra MAX-190, Molecular Devices, Sunnyvale, CA, USA). A standard curve was prepared using dilutions of purified MMP-9 and MMP-9 concentrations in the samples were determined. Detection limit for MMP-9 was 0.1 ng/mL. The inter- and intra-assay coefficients of variation were 8.4 and 11.5%, respectively [11]. Myocardial damage was estimated by measurements of troponin I release. Troponin I was determined immunoenzymatically using a technique based on sandwich ELISA (Boehringer Mannheim, Mannheim 68305, Germany). Coefficients of variation were 2.2% and 5.9% for intra-assay and inter-assay variabilities, respectively. Blood samples for troponin I assessment were taken every 8 h during the first day.

### 2.3. End-Points

Primary endpoint was the incidence of death or readmission due to heart failure (defined as worsening of heart failure symptoms that required admission) at 2 years of follow-up. Secondary endpoint was levels of MMP-9 after the primary percutaneous coronary intervention.

### 2.4. Statistical Analysis

Quantitative variables that had a normal distribution were presented as mean ± SD; others were presented as median (interquartile range). To test if quantitative variables had a normal distribution, Shapiro–Wilk test and Q-Q plots were used. We compared quantitative variables with normal distribution using *t*-test for equal or unequal variances. Variances were compared using the robust Levene test. Skewed variables were compared using the nonparametric Mann–Whitney U test. Categorical variables were compared using the Fisher exact test except for variables with several categories that could be ordered. For this last case, the trend test was used.

For analyzing the primary end-point (clinical outcomes during the follow-up), we used the restricted mean survival time (RMST), which does not depend on number of events or proportionality of hazards. The RMST is the average time free from an event up until a milestone time point, a numeric expression of the area under the Kaplan–Meier survival curve. RMST of both groups can be compared, allowing the estimation of the difference of time without the event between arms. For example, a difference in RMST of 10 days would indicate that the treatment group lived on average 10 days longer than the control group [12,13]. Survival curves were constructed by the Kaplan–Meier method. Log-rank test and the hazard ratio (HR) (using a univariate Cox model) were also calculated.

For the secondary end-point, MMP-9 levels were directly compared between both groups. Despite randomization, some baseline characteristics were different between groups. As sensitivity analysis, we controlled for all baseline variables that were statistically different between arms. To do that, we created a multivariate linear regression model where MMP-9 was the dependent variable, melatonin/placebo was the exposure variable, and the independent variables were those that had statistically significant differences between both groups. Diagnostics of the model were tested. We presented the coefficient with the 95% confidence intervals (CI). Data was analyzed according to the ‘intention to treat’ principle.

## 3. Results

**Figure 1:**
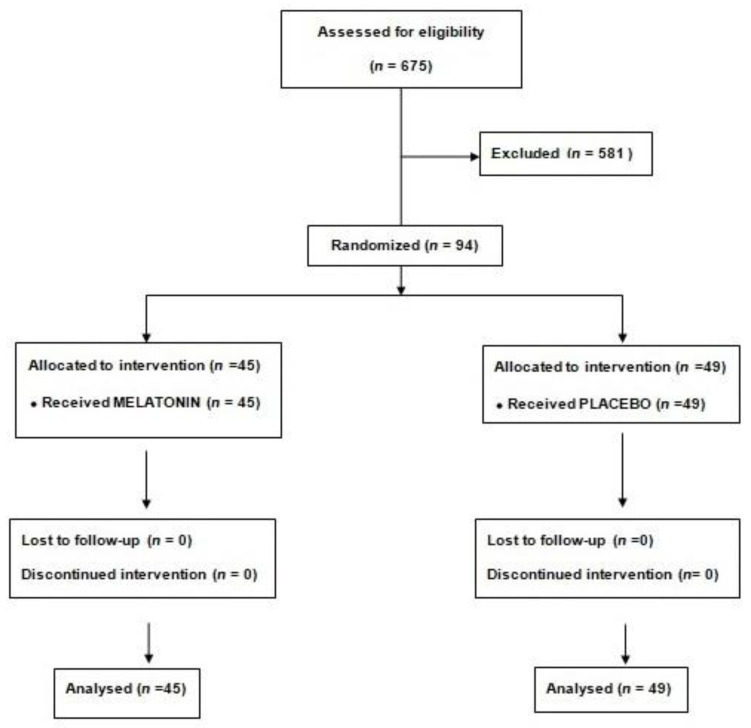
Consort diagram.

Ninety-four patients were randomized to melatonin (45) or placebo (49) (Figure 1).

Median age was 62.4 (55.7–75.9) and 43 (45.7%) were women. There was no crossover between treatments. All patient characteristics are shown in Table 1. Despite randomization, weight, hemoglobin levels and number of diseased coronary vessels had statistically significant differences between groups. The most frequent location for the infarction was inferior in 48 patients (51.1%), the peak troponin was 212 (181–262) and 66 (70.2%) patients had one-vessel disease. There were no statistically significant differences in treatments at discharge between groups.

### 3.1. Primary End-Point

**Figure 2:**
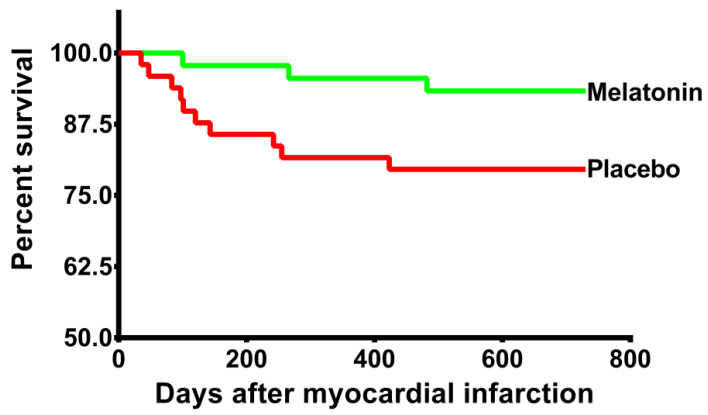
**Kaplan–Meier curves:** The Kaplan–Meier curves show the cumulative incidence of the primary endpoint, which was a composite of death and heart failure readmission at 2 years.

No patient was lost to follow-up. All censored observations reached 2 years of follow-up. During the two years of follow-up, 13 patients (13.8%) exhibited the combined event (3 deaths and 10 readmissions due to heart failure). Three patients in the melatonin group and 10 in the placebo group manifested these events. The mean time until a clinical outcome event occurred was 699.2 days and 611.8 days for the melatonin and placebo group respectively. RMST difference was 87.5 days (*p* = 0.02). HR = 0.3 (95% CI 0.08–1.08; *p* = 0.06). Controlling for hemoglobin and number of diseased vessels, the model showed HR = 0.27 (95% CI 0.08–0.97; *p* = 0.046). Log-rank test 0.04. Kaplan–Meier survival curves are shown in Figure 2.

### 3.2. Secondary End-Point

**Figure 3:**
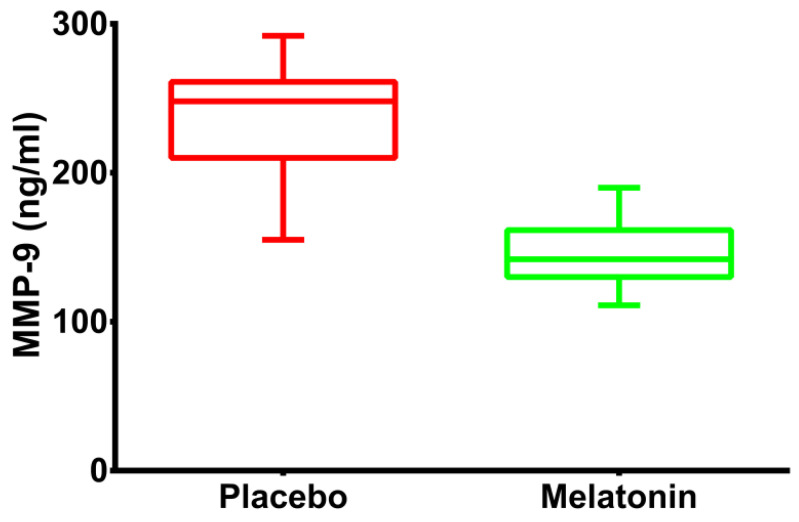
**Changes in the levels of MMP-9:** Boxplot graphs illustrating the levels of MMP-9 in the 2 treatment arms in patients with acute myocardial infarction undergoing primary percutaneous coronary intervention.

Compared with the placebo, MMP-9 levels were reduced in the melatonin group (248 ng/mL (211–259) vs. 142 ng/mL (130–161) *p* < 0.001). The distribution of this molecule between groups can be observed in Figure 3. After controlling for weight, hemoglobin and number of diseased coronary vessels, the treatment with melatonin reduced the levels of MMP-9 to 90 ng/mL (95% CI 77.3–102.6).

## 4. Discussion

The results of this pilot study indicate that treatment with intravenous melatonin in patients with AMI undergoing primary percutaneous coronary intervention is associated with a reduced incidence of death or readmission due to heart failure. This effect was accompanied by a substantial treatment-related difference in MMP-9 levels, a marker of AMI remodeling response.

MMP are a family of zinc-dependent endopeptidases (collagenase type IV) which are synthesized as zymogens. Of particular interest in the heart is MMP-9, which is found in cardiac myocyte, cardiac fibroblasts, and endocardial cells [14]. MMP-9 is well recognized for its proteolytic action on extracellular matrix proteins and its involvement in long-term remodeling processes that occur in pathological events such as atherosclerosis and heart failure [14]. Recently, Lan et al. demonstrated in an animal study that treatment with melatonin protected the heart from IRI by reducing the expression levels of MMP-9 [15]. To our knowledge, no study has assessed the effect of melatonin on levels of MMP-9 in patients with AMI. In our study, MMP-9 levels decreased in the melatonin group compared with the control group. Therefore, pretreatment with melatonin can mitigate inflammation [16].

AMI is one of the biggest challenges for the health systems of western countries. Survival from myocardial infarction has improved considerably because of early, effective treatment, but myocardial infarction leads to chronic heart failure in many cases, exerting a huge global healthcare burden on a country’s economic resources. Despite timely reperfusion with primary percutaneous coronary intervention, mortality and morbidity following AMI remain significant, with 7% death and 22% hospitalization for heart failure at one year [17]. The social impact of ischemic heart disease is enormous, not just because of the resulting mortality, but also because of lower quality of life and the high economic costs involved. New cardioprotective strategies are needed to attenuate the damaging effects of IRI and thereby prevent adverse left ventricular remodeling, improve survival and reduce heart failure [18]. Our study showed at 2 years of follow-up that patients treated with melatonin lived without hospital admission for heart failure, on average, 90 days longer than those treated with placebo.

Recently, preclinical or clinical data regarding the role of melatonin in myocardial IRI has been reported in two meta-analyses [7,17]. Mao et al. published a meta-analysis of animal studies [7], demonstrating that pretreatment with melatonin was associated with a significantly lower infarct size in comparison with placebo in myocardial IRI. On the other hand, a recent meta-analysis by Dominguez-Rodriguez and colleagues on randomized controlled trials in humans showed that melatonin administration improved left ventricular function and reduced troponin levels, which is consistent with a possible cardioprotective effect of this molecule [17]. It is possible that melatonin plays a role in reducing IRI due to a complex pathophysiological interplay in the re-perfused AMI [4].

The limitations of this study should be recognized. First, the MMP-9 activity is a time-dependent process which increases significantly at 2 to 24 h after reperfusion [18]. In our pilot study, we only measured the MMP-9 just after completion of the primary percutaneous coronary intervention. Second, a coronary sinus sample for MMP-9 levels would have been much more valuable than a systemic value. Third, we decided to create this pilot study so that it may be used in the future to calculate the adequate sample size for a large randomized clinical trial. Fourth, data on the left ventricular ejection fraction in the follow up was not collected. Finally, a limited number of patients were enrolled in this study. Despite randomness, both groups could be unbalanced and large-scale, double-blind, placebo-controlled clinical studies will be required to confirm this efficacy. Nevertheless, our results suggest that early administration of melatonin, within 3 h of symptoms, is associated with a better outcome in patients with AMI undergoing primary percutaneous coronary intervention, and point to a novel adjuvant therapy strategy for recovery, at least in part, in the infarcted zone.

## Figures and Tables

**Table 1:** Demographic, clinical, and procedural patient characteristics per randomized group.

Variable	Placebo (*n* = 49)	Melatonin (*n* = 45)	*p*-Value
Age (years)	60.5 (54.9–69.7)	60.4 (57–77.3)	0.11
Women; *n* (%)	26 (53.1%)	17 (37.8%)	0.15
Diabetes; *n* (%)	7 (14.3%)	4 (8.9%)	0.53
Hypertension; *n* (%)	28 (57.1%)	23 (51.1%)	0.68
Dyslipidemia; *n* (%)	29 (59.2%)	23 (51.1%)	0.53
Weight (kg)	61.7 ± 8.9	67 ± 10	0.01
Height (m)	1.7 ± 0.1	1.7 ± 0.1	0.31
Smoking habit; *n* (%)	28 (57.1%)	23 (51.1%)	0.68
Total cholesterol (mg/dL)	174 (153–213)	177 (153–208)	0.87
LDL cholesterol (mg/dL)	104 (82–138)	108 (86–136)	0.94
Hemoglobin (g/dL)	13.5 (12–14.8)	14.4 (13.1–15.2)	0.049
***Cardiac characteristics***			
Infarction location			0.7
Anterior; *n* (%)	23 (46.9%)	18 (40%)	
Inferior; *n* (%)	23 (46.9%)	25 (55.6%)	
Lateral; *n* (%)	3 (6.1%)	2 (4.4%)	
Pain-to-reperfusion time (minutes)	137.4 ± 18.4	134.6 ± 19.0	0.46
Left ventricular ejection fraction (%)	56.1 (50–60)	57.5 (53.3–63.8)	0.24
Troponin I peak (pg/mL)	204 (176–239)	219 (188–277)	0.23
Number of diseased vessels			0.03
1 vessel disease; *n* (%)	30 (61.2%)	36 (80%)	
2 vessel disease; *n* (%)	17 (34.7%)	9 (20%)	
3 vessel disease; *n* (%)	2 (4.1%)	0 (0%)	
***Treatment at discharge***			
Antiplatelet drugs; *n* (%)	49 (100%)	45 (100%)	1
Angiotensin converting enzyme inhibitors; *n* (%)	40 (81.6%)	30 (66.7%)	0.11
Beta blockers; *n* (%)	47 (95.9%)	42 (93.3%)	0.67
Statins; *n* (%)	49 (100%)	45 (100%)	1

Continuous variables are summarized as mean ± standard deviation or median with interquartile range. Categorical variables are presented as count (percentage).

## Data Availability

The data that support the findings of this study are available on request from the corresponding author, A.D.-R. The data are not publicly available due to their containing information that could compromise the privacy of research participants.

## References

[1] Hausenloy D.J., Garcia-Dorado D., Bøtker H.E., Davidson S.M., Downey J., Engel F.B., Jennings R., Lecour S., Leor J., Madonna R. (2017). Novel targets and future strategies for acute cardioprotection: Position Paper of the European Society of Cardiology Working Group on Cellular Biology of the Heart. Cardiovasc. Res..

[2] Hausenloy D., Barrabés J.A., Bøtker H.E., Davidson S., Di Lisa F., Downey J., Engstrom T., Ferdinandy P., Carbrera-Fuentes H.A., Heusch G. (2016). Ischaemic conditioning and targeting reperfusion injury: A 30 year voyage of discovery. Basic Res. Cardiol..

[3] Jacob-Ferreira A.L., Schulz R. (2013). Activation of intracellular matrix metalloproteinase-2 by reactive oxygen-nitrogen species: Consequences and therapeutic strategies in the heart. Arch. Biochem. Biophys..

[4] Dominguez-Rodriguez A., Abreu-Gonzalez P., Chen Y.H. (2019). Cardioprotection and effects of melatonin administration on cardiac ischemia reperfusion: Insight from clinical studies. Melatonin Res..

[5] Dominguez-Rodriguez A., Abreu-Gonzalez P., Avanzas P. (2012). The role of melatonin in acute myocardial infarction. Front. Biosci..

[6] Dominguez-Rodriguez A., Abreu-Gonzalez P., Sanchez-Sanchez J.J., Kaski J.C., Reiter R.J. (2010). Melatonin and circadian biology in human cardiovascular disease. J. Pineal Res..

[7] Mao Z.J., Lin H., Xiao F.Y., Huang Z.Q., Chen Y.H. (2020). Melatonin against Myocardial Ischemia-Reperfusion Injury: A Meta-analysis and Mechanism Insight from Animal Studies. Oxid. Med. Cell. Longev..

[8] Dominguez-Rodriguez A., Abreu-Gonzalez P., de la Torre-Hernandez J.M., Consuegra-Sanchez L., Piccolo R., Gonzalez-Gonzalez J., Garcia-Camarero T., Del Mar Garcia-Saiz M., Aldea-Perona A., Reiter R.J. (2017). Usefulness of Early Treatment With Melatonin to Reduce Infarct Size in Patients With ST-Segment Elevation Myocardial Infarction Receiving Percutaneous Coronary Intervention (From the Melatonin Adjunct in the Acute Myocardial Infarction Treated With Angioplasty Trial). Am. J. Cardiol..

[9] Ibanez B., James S., Agewall S., Antunes M.J., Bucciarelli-Ducci C., Bueno H., Caforio A.L.P., Crea F., Goudevenos J.A., Halvorsen S. (2018). 2017 ESC Guidelines for the management of acute myocardial infarction in patients presenting with ST-segment elevation: The Task Force for the management of acute myocardial infarction in patients presenting with ST-segment elevation of the European Society of Cardiology (ESC). Eur. Heart J..

[10] Fujimoto N., Hosokawa N., Iwata K., Shinya T., Okada Y., Hayakawa T. (1994). A one-step sandwich enzyme immunoassay for inactive precursor and complexed forms of human matrix metalloproteinase 9 (92 kDa gelatinase/type IV collagenase, gelatinase B) using monoclonal antibodies. Clin. Chim. Acta.

[11] Dominguez-Rodriguez A., Abreu-Gonzalez P., Garcia-Gonzalez M.J., Kaski J.C. (2008). High serum matrix metalloproteinase-9 level predict increased risk of in-hospital cardiac events in patients with type 2 diabetes and ST segment elevation myocardial infarction. Atherosclerosis.

[12] Kim D.H., Uno H., Wei L.J. (2017). Restricted Mean Survival Time as a Measure to Interpret Clinical Trial Results. JAMA Cardiol..

[13] Royston P., Parmar M.K. (2013). Restricted mean survival time: An alternative to the hazard ratio for the design and analysis of randomized trials with a time-to-event outcome. BMC Med. Res. Methodol..

[14] Lalu M.M., Pasini E., Schulze C.J., Ferrari-Vivaldi M., Ferrari-Vivaldi G., Bachetti T., Schulz R. (2004). Ischaemia-reperfusion injury activates matrix metalloproteinases in the human heart. Eur. Heart J..

[15] Lan H., Su Y., Liu Y., Deng C., Wang J., Chen T., Jules K.E.D., Masau J.F., Li H., Wei X. (2019). Melatonin protects circulatory death heart from ischemia/reperfusion injury via the JAK2/STAT3 signalling pathway. Life Sci..

[16] Chitimus D.M., Popescu M.R., Voiculescu S.E., Panaitescu A.M., Pavel B., Zagrean L., Zagrean A.-M. (2020). Melatonin’s Impact on Antioxidative and Anti-Inflammatory Reprogramming in Homeostasis and Disease. Biomolecules.

[17] Domínguez-Rodríguez A., Abreu-González P., Báez-Ferrer N., Reiter R.J., Avanzas P., Hernández-Vaquero D. (2021). Melatonin and Cardioprotection in Humans: A Systematic Review and Meta-Analysis of Randomized Controlled Trials. Front. Cardiovasc. Med..

[18] Kunugi S., Shimizu A., Kuwahara N., Du X., Takahashi M., Terasaki Y., Fujita E., Mii A., Nagasaka S., Akimoto T. (2010). Inhibition of matrix metalloproteinases reduces ischemia-reperfusion acute kidney injury. Lab. Investig..

